# Correction: Perceptions and practices related to birthweight in rural Bangladesh: Implications for neonatal health programs in low- and middle-income settings

**DOI:** 10.1371/journal.pone.0229165

**Published:** 2020-02-07

**Authors:** The PLOS ONE Staff

## Notice of Republication

An incomplete, earlier version of this article was published in error. The publisher apologizes for the error. This article was republished on January 24, 2020, to correct for this error. Please download the article again to view the correct version. The originally published, uncorrected article and the republished, corrected article are provided here for reference.

## Supporting information

S1 File Originally published, uncorrected article. (PDF)

S2 File Republished, corrected article. (PDF)
