# Supplementary material for: Perceptions and practices related to birthweight in rural Bangladesh: Implications for neonatal health programs in low- and middle-income settings
Source: PLoS One. 2019 Dec 30;14(12):e0221691. doi: 10.1371/journal.pone.0221691 (PMC6936797; doi:10.1371/journal.pone.0221691)
Supplement: S3 File — (PDF) [file pone.0221691.s003.pdf]

## Guideline for focus group discussions (Husbands)

***Perceptions and practices related to birthweight in rural Bangladesh***

- How does a mother's health impact the growth of a baby?
- How do you think a baby (fetus) grows up inside the mother's womb?
- What is the relationship between nourishment of a baby when it is still in its mother's womb and the food consumed by its mother?
- Did/do you advise your wife to make any changes to her dietary practice in consideration for the nutrition and development of the fetus? If yes, can you explain what kind of changes are those?
- What are the first few questions people in your area usually ask immediately after birth of a baby?
- How would you define a healthy baby? What are the criteria of a healthy baby?
- How can you or your family members ensure a healthy baby is born?
- What is your understanding about birthweight?
- What is the necessity of weighing an infant's birthweight?
- In your opinion is there any relationship between birthweight and health status of a baby? If yes, please explain.
- Are you familiar with the weighing scale? If yes where did you see it? Please explain.
- What do you think within what time of birth, birthweight should be taken?
- Where did you get any information about birthweight?
- Do people in your area talk about birthweight and/or birth size? What sort of things do they talk about?
- Have you thought about the birthweight and/or birth size of your baby before it was born?
- Have you ever discussed with your wife about your baby's birthweight and/or birth size?
- What was your baby's birthweight? Was your baby weighted at its birth? If not weighed at birth, how did you know your baby's birthweight?
- How would you describe your baby's birthweight birth size?
- Did any of your family members ever mention about the possible/expected weight and/or size of the baby at its birth?

- Is there any expected ideal weight or size of a newborn at its birth? what was your expectation about your baby's birthweight or birth size?
- Can you classify a baby's birthweight or birth size? Please explain.
- What are the terms you and people in your area use to describe 'birthweight', 'low birthweight' and 'high birthweight'?
- Please describe a low birthweight /high birthweight/normal birthweight baby.
- Which birthweight do you consider an ideal birthweight? Why?
- What are the causes of low birthweight and high birthweight?
- What are the consequences of low birthweight and high birthweight?
- How can you or other family members ensure that a baby is born with an ideal birthweight?
- What can a mother do in her pregnancy to avoid a low birthweight/ or small birth size baby?
- What do you do usually if a baby is born with a low birthweight /small size?
- Is there any treatment needed for a low birthweight /small size baby? In your opinion what should be the treatment for an infant with a low birthweight and high birthweight? Please explain.
