# Supplementary material for: Perceptions and practices related to birthweight in rural Bangladesh: Implications for neonatal health programs in low- and middle-income settings
Source: PLoS One. 2019 Dec 30;14(12):e0221691. doi: 10.1371/journal.pone.0221691 (PMC6936797; doi:10.1371/journal.pone.0221691)
Supplement: S4 File — (PDF) [file pone.0221691.s004.pdf]

Guideline for focus group discussions (Husbands)

Perceptions and practices related to birthweight in rural Bangladesh

- আপনার কি মনে হয় কিভাবে একটা গর্ভজাত শিশু তার মা এর গর্ভে কিভাবে বেড়ে উঠে?
- আপনি কি মনে করেন যে মা এর খাদ্যাভ্যাস এর সাথে তার গর্ভজাত শিশুর বেড়ে উঠার কোন সম্পর্ক আছে?
  - কিভাবে মা এর খাদ্যাভ্যাস তার গর্ভজাত শিশুর বেড়ে উঠাকে প্রভাবিত করে?
- আপনি কি আপনার স্ত্রীকে তার গর্ভজাত শিশুর পুষ্টি ও বেড়ে উঠার জন্য তার খাদ্যাভ্যাস এ কোন পরিবর্তন আনার কথা বলেছেন? যদি হ্যাঁ হয়, কিরকম পরিবর্তন সেগুলো?
- একটি শিশুর জন্মের পরপরই সাধারনত কোন প্রশ্নগুলো করা হয় আপনার এলাকাতে?
- একটি সুস্থ নবজাতক বলতে আপনি কি বুঝেন? একটি সুস্থ নবজাতকের বৈশিষ্ট্য গুলো কি কি?
- আপনি বা আপনার পরিবারের বাকি সদস্যরা কিভাবে একটি সুস্থ শিশুর জন্ম নিশ্চিত করতে পারে?
- একটি শিশুর জন্মের সময় যে ওজন মাপা হয় সে সম্পর্কে আপনার মতামত কি?
- জন্মের সময় শিশুর ওজন মাপার প্রয়জনিয়তা কি?
- জন্মের সময় শিশুর ওজন এর সাথে শিশুর সুস্থতার কোন সম্পর্ক আছে কি? যদি থাকে বিস্তারিত বলুন।
- আপনি কি ওজন মাপার যন্ত্রের সাথে পরিচিত?
- আপনার কি মনে হয় জন্মের ওজন মাপার সঠিক সময় কখন?
- আপনি কি কোথাও থেকে শিশুর জন্মের সময়ের আকার বা ওজন এর ব্যাপারে কোন তথ্য পেয়েছেন ?
- আপনার এলাকাতে কেউ কি শিশুর জন্মের সময়ের ওজন অথবা আকার নিয়ে কথা বলে? বিস্তারিত বলুন।
- আপনি কি আপনার আগত শিশুর জন্মের ওজন অথবা আকার নিয়ে কিছু চিন্তা করেছেন/করেছিলেন?
- আপনি কি আপনার আগত শিশুর জন্মের আকার অথবা ওজন কেমন হতে পারে তা নিয়ে
- আপনি কি আপনার শিশুর জন্মের আগে তার জন্মের সময়ের আকার অথবা ওজন কেমন হতে পারে তা নিয়ে আপনার স্ত্রীর সাথে আলোচনা করেছিলেন/করেছেন?
- আপনার শিশুর জন্মের সময় ওজন কত ছিল? আপনার শিশুর জন্মের ওজনটা কি মাপা হয়েছিল? যদি না হয়ে থাকে তাহলে কিভাবে আপনি আপনার শিশুর ওজন জেনেছিলেন?
- আপনার শিশুর জন্মের সময় ওজন এবং আকার সম্পর্কে বর্ণনা করুন।
- আপনার পরিবারের কোন সদস্য কি আপনার আগত শিশুর জন্মের আকার অথবা ওজন কেমন হতে পারে তা নিয়ে কখনও কিছু আলোচনা করেছে?
- আপনার কি আপনার শিশুর জন্মের সময়ের ওজন নিয়ে কোন প্রত্যাশা আছে/ছিল?
- আপনার মতে একটি শিশুর জন্মের সময় ওজন কয় ধরনের হতে পারে? বিস্তারিত বলুন।
- আপনার এলাকাতে শিশুর জন্মের ওজন, কম ওজন এবং বেশি ওজন বুঝাতে কোন শব্দগুলো ব্যাবহার করা হয়?
- শিশুর জন্মের কম ওজন/বেশি ওজন/ সাভাবিক ওজন বলতে কি বুঝেন? বর্ণনা করুন।
- আপনার কি মনে হয় একটি শিশুর জন্মের আদর্শ ওজন অথবা আকার কিরকম হওয়া উচিত?
- একটি শিশুর কম ওজন/ বেশি ওজন হওয়ার কারণগুলো কি হতে পারে?
- একটি শিশুর কম ওজন/ বেশি ওজন হওয়ার পরিণতিগুলো কি হতে পারে?
- আপনি বা আপনার পরিবারের বাকি সদস্যরা কিভাবে একটি শিশুর সাভাবিক জন্মের ওজন নিশ্চিত করতে পারেন?
- আপনার মতে একটি গর্ভবতী মা গরভাবস্থায় কি কি করলে একটি শিশুর কম ওজন/ ছোট আকার প্রতিরোধ করা যায়?
- একটি ছোট অথবা কম ওজনের শিশুর জন্ম হলে আপনারা সাধারণত কি করেন?
- আপনার কি মনে হয় একটি ছোট অথবা কম ওজনের শিশুর কি কোন যত্ন নেওয়া দরকার? আপনার মতে কিভাবে একটি ছোট অথবা কম ওজনের শিশুর যত্ন নেওয়া যেতে পারে?
