# Supplementary material for: Perceptions and practices related to birthweight in rural Bangladesh: Implications for neonatal health programs in low- and middle-income settings
Source: PLoS One. 2019 Dec 30;14(12):e0221691. doi: 10.1371/journal.pone.0221691 (PMC6936797; doi:10.1371/journal.pone.0221691)
Supplement: S5 File — (PDF) [file pone.0221691.s005.pdf]

Guideline for key-informant interviews (Health care providers)

Perceptions and practices related to birthweight in rural Bangladesh

- Do you talk to pregnant women or their families about the growth of the baby?
- What do you usually advise the women in their pregnancies in relation to the wellbeing of their upcoming baby?
- Do you advise the pregnant women to make any changes to their dietary practice in consideration for the nutrition and development of the fetus? What kind of changes are those? Please explain.
- Do you talk to the pregnant women about their babies' birthweight? If yes, please explain.
- Do pregnant women or their families in your area talk about the birthweight or birth size?
- What do you think about the families' perception of their babies' birthweight?
- Is there any expected ideal weight or size of a newborn in your community at its birth?
- What are the terms people in your area use to describe 'birthweight', 'low birthweight' and 'high birthweight'?
- Do families in your area recognise the risk associated with a low birthweight infant?
- Is there any preventive practice a pregnant woman or her family does to ensure a healthy baby is born with right weight and size?
- If a low birthweight baby is born in, what do the families of your area usually do? Do they report to you or any other health care provider? Do they perform any care practices at home for the LBW baby?
- As a health care provider, do you have any recommendations for improving community perceptions of birthweight? If yes, what are those?
