# Supplementary material for: Perceptions and practices related to birthweight in rural Bangladesh: Implications for neonatal health programs in low- and middle-income settings
Source: PLoS One. 2019 Dec 30;14(12):e0221691. doi: 10.1371/journal.pone.0221691 (PMC6936797; doi:10.1371/journal.pone.0221691)
Supplement: S6 File — (PDF) [file pone.0221691.s006.pdf]

Guideline for key-informant interviews (Health care providers)

Perceptions and practices related to birthweight in rural Bangladesh

- আপনি কি গর্ভবতী মহিলা অথবা তাদের পরিবারের সদস্যদের সাথে গর্ভজাত শিশুর বেড়ে উঠার ব্যাপারে কথা বলেন?
- আপনি কি গর্ভবতী মহিলাদের তাদের শিশুর পুষ্টি ও বেড়ে উঠার জন্য তার খাদ্যাভ্যাস এ কোন পরিবর্তন আনার কথা আলোচনা করেন? যদি হ্যাঁ হয়, কিরকম পরিবর্তন সেগুলো?
- আপনি কি আপনার গর্ভবতী মহিলাদের সাথে তাদের শিশুর জন্মের সময়ের ওজন নিয়ে আলোচনা করেন?
- আপনার এলাকাতে গর্ভবতী মহিলারা তাদের আগত শিশুর জন্মের সময়ের আকার অথবা ওজন নিয়ে কি কথা বলে?
- আপনার কি মনে হয় আপনার এলাকার গর্ভবতী মহিলাদের শিশুর জন্মের সময়ের আকার অথবা ওজন এর ব্যাপারে তাদের ধারনা কেমন?
- তাদের কি শিশুর জন্মের আদর্শ আকার অথবা ওজন নিয়ে কোন প্রত্যাশা থাকে?
- আপনার এলাকাতে শিশুর জন্মের ওজন, কম ওজন এবং বেশি ওজন বুঝাতে কোন শব্দগুলো ব্যাবহার করা হয় সাধারনত?
- আপনার কি মনে হয় আপনার এলাকায় পরবিবাররা কম ওজনের শিশুর শারীরিক সমস্যাগুলোর সাথে কতটুকু পরিচিত?
- আপনার এলাকার গর্ভবতী মা রা গর্ভাবস্থায় শিশুর কম ওজন/ ছোট আকার প্রতিরোধ করার জন্য কি কিছু করে?
- একটি কম ওজন অথবা ছোট শিশু জন্মগ্রহন করলে আপনার এলাকার শিশুর পরিবাররা সাধারণত কি করে? তারা কি আপনাকে বা অন্য কোন স্বাস্থ্য সেবককে এই ব্যাপারে অবহিত করে? তারা কি তাদের শিশুর বাসায় কোন যত্ন নেয়?
- আপনি যেহেতু এই এলাকাতে মা ও শিশু দের চিকিৎসাসেবা দান করেন, আপনার কি কোন পরামর্শ আছে কিভাবে এই এলাকার মা ও তার পরিবারদের শিশুর জন্মের সময়ের ওজন সম্পর্কিত ধারনার উন্নতি করা যায়?
