# Supplementary material for: Correction: Perceptions and practices related to birthweight in rural Bangladesh: Implications for neonatal health programs in low- and middle-income settings
Source: PLoS One. 2020 Feb 7;15(2):e0229165. doi: 10.1371/journal.pone.0229165 (PMC7006918; doi:10.1371/journal.pone.0229165)
Supplement: S2 File — (PDF) [file pone.0229165.s002.pdf]

RESEARCH ARTICLE

Perceptions and practices related to birthweight in rural Bangladesh: Implications for neonatal health programs in low- and middle-income settings

Monjura Khatun Nisha[1]*, Camille Raynes-Greenow[1], Aminur Rahman[2], Ashraful Alam[1]

1 Sydney School of Public Health, The University of Sydney, NSW, Sydney, Australia, **2** Health Systems and Population Studies Division, icddr,b, Dhaka, Bangladesh

* mnis2091@uni.sydney.edu.au

Check for updates

OPEN ACCESS

Citation: Nisha MK, Raynes-Greenow C, Rahman A, Alam A (2019) Perceptions and practices related to birthweight in rural Bangladesh: Implications for neonatal health programs in low- and middle-income settings. PLoS ONE 14(12): e0221691. https://doi.org/10.1371/journal.pone.0221691

Editor: Emma Sacks, Johns Hopkins School of Public Health, UNITED STATES

Received: November 22, 2018

Accepted: December 6, 2019

Published: December 30, 2019

Copyright: © 2019 Nisha et al. This is an open access article distributed under the terms of the Creative Commons Attribution License, which permits unrestricted use, distribution, and reproduction in any medium, provided the original author and source are credited.

Data Availability Statement: Due to ethical restrictions related to protecting study participants' privacy, data access is subject to an application following icddr,b data access policy (https://www.icddrb.org/dmdocuments/icddrb%20Data%20Access%20Policy.pdf). Interested researchers may contact Ms Armana Ahmed (Head, Research Administration, icddr,b) (aahmed@icddrb.org) with further inquiries related to data access.

Funding: This study was partly supported through funding received from the Sydney School of Public

Abstract

Background

Globally, low birthweight (LBW) infants (<2,500 grams) contribute up to 80% of neonatal mortality. In Bangladesh, approximately 62% of all births occur at home and therefore, weighing newborns immediately after birth is not feasible. Thus, estimates of birthweight in Bangladesh are mostly obtained based on maternal perception of the newborn's birth size. Little is known about how birthweight is perceived in rural communities, and whether families associate birthweight with newborn's health status. Our objective was to explore families' perceptions of newborn's birthweight, preventive practices to LBW, and care practices for a LBW newborn in rural Bangladesh.

Methods

We conducted a qualitative study in two rural settings of Bangladesh, including 32 in-depth interviews (11 with pregnant women, 12 with recently delivered women, 4 with husbands whose wives were pregnant or had a recent birth, 5 with mothers-in-law whose daughters-in-law were pregnant or had a recent birth), 2 focus group discussions with husbands and 4 key-informant interviews with community health workers. We used thematic analysis to analyse the data.

Results

Most participants did not consider birthweight a priority for assessing a newborn's health status. Recognition of different categories of birthweight was subjective and often included several physical descriptors including birth size of the newborn. LBW was not considered as a criterion of a newborn's illness unless the newborn appeared unwell. Maternal poor nutrition, inadequate diet in pregnancy, anaemia, illnesses during pregnancy, short stature, twin births and influence of supernatural spirit were identified as the major causes of LBW. Women's preventive practices to LBW or small newborns were predominantly constrained by a

Health, the University of Sydney. We are grateful to Sydney Medical School, The University of Sydney for funding MKN's PhD (Early Career Researcher Postgraduate Scholarship). CRG is funded by the National Health and Medical Research Council Career Development Award #1087062. The funders had no role in study design, data collection and analysis, decision to publish, or preparation of the manuscript.

Competing interests: The authors have declared that no competing interests exist.

lack of awareness of birthweight and fear of caesarean section. As an effort to avoid caesarean section during birth, several women tended to perform potentially harmful practices in order to give birth to a LBW or small size newborn; such as avoiding nutritious food and decreasing food intake in pregnancy. Common practices to treat a LBW or small newborn who appeared ill included breastfeeding, feeding animal milk, feeding sugary water, feeding formula, oil massage, keeping the small newborn warm and seeking care from formal and informal care providers including a spiritual leader. Maternal lack of decision-making power, financial constraint, home birth and superstition were the major challenges to caring for a LBW newborn.

Conclusion

Birthweight was not well-understood in the rural community, which highlighted substantial challenges to the prevention and care practices of LBW newborns. Community-level health education is needed to promote awareness related to the recognition of birthweight in rural settings.

Background

Birthweight is an important determinant of perinatal (perinatal period commences at 22 completed weeks of gestation and ends seven completed days after birth) [1], neonatal (infants between the age of 0 and 28 days) [2] and infant (0 to 1 year of age) [1] mortality and morbidity [2]. Low birthweight (LBW) which is defined by the World Health Organization as a birthweight of less than 2,500 grams (g) regardless of gestational age [1] occurs either due to preterm birth (born early) or small-for-gestational-age [3]. In resource-poor settings, where valid estimates of gestational age are often not available because of late and infrequent access to prenatal care, inadequate documentation of the date of the last menstrual period and unavailability of early ultrasound examination, "LBW" is preferred by epidemiologists due to its ability to be measured in more precision compared with preterm birth or small-for-gestational-age [3]. Infants born with LBW account for 60-80% of all neonatal deaths [4, 5]. The risk for neonatal death is approximately three times greater for LBW infants than for infants with a birthweight of more than 2,500 g and the risk increases up to 8 times as birthweight decreases [6]. Further, LBW is associated with a higher risk of morbidity [7], stunting [8] and several long-term adverse consequences, including cognitive and behavioural development delays in children [9]. The impact of LBW continues into adulthood and LBW increases the risk of chronic diseases (e.g. cardiovascular disease and diabetes) in adults [10–13]. Recognising the importance of LBW, in 2012, the World Health Assembly set a target to reduce LBW by 30% by 2025, which requires a 3% relative reduction per year between the years 2012 and 2025 [14].

Globally, every year an estimated 20 million (15.5%) infants are born with LBW [4, 5], approximately 97% of these births occur in low- and middle-income countries (LMICs) [15], with the highest proportion in the South Asian region (28%) [16, 17]. Approximately 66% of infants in South Asia are not weighed at birth due to birthing at home, instead, maternal perception of birth size is often used as a source of birthweight information for estimating the proportion of LBW infants in these South Asian countries [16, 18, 19]. Several studies reported that the concordance between birth size and birthweight may vary by an under or over-estimation [20–25], which suggests that a mother's perception of her newborn's birth size may be

influenced by other concurrent factors. In a previous study using Demographic Health Survey data from three countries, Channon attributed the discrepancies between birth size and birthweight to the infant sex, survivability of the infant and societal or community influences on maternal perception of birth size [26]. Understanding community perceptions on birthweight is crucial for newborn survival, as care-seeking for any newborn illness depends on the recognition and perceptions of the illness [27, 28]. Little is known about how birthweight is perceived and recognised in rural communities, and if it is considered an important determinant of a newborn's health. Darmstadt et al. reported that there was little perceived need to be acquainted with the newborn's birthweight in rural India [29]. Further, a recent study conducted in Malawi identified community perception as the major constraint of seeking care for LBW newborns in rural communities [30]. In Bangladesh, estimates of birthweight are mostly obtained based on maternal recall and perceptions of the newborn's birth size, as approximately 62% of all births occur at home and weighing newborns immediately after birth is not feasible [31]. Moreover, a major proportion (69%) of these home births occur in rural areas of Bangladesh, where LBW is prevalent [31, 32], and overall health care-seeking for newborns is low due to poor knowledge of risk behaviours and limited access to health care [33]. Whether rural communities in Bangladesh associate birthweight with newborn's health status and how this impacts the care-seeking practices for a LBW newborn are unknown. Therefore, our objective was to explore families' perceptions of newborn's birthweight including local meaning and causes of different categories of birthweight in rural Bangladesh. We further aimed to explore families' preventive practices to LBW, care practices for a LBW newborn and barriers to care for a LBW newborn at the community level.

Methods

Study design

We used a qualitative study design involving in-depth interviews (IDI), key informant interviews (KII), and focus group discussions (FGD) to explore the perceptions and practices around birthweight among women and their families.

Study sites, study population and selection of participants

We used purposeful sampling for recruiting the participants in two rural settings within Sakhipur upazila of Tangail district in the central part of Bangladesh. Eligible women were pregnant or recently delivered who gave birth within1 year. As older women in the family have an influence on younger women's perceptions and practices around pregnancy and childbirth, we also included mothers-in-law of the pregnant or recently delivered women to generate information on the older generation's views of birthweight. Husbands whose wives were pregnant or had a recent birth were also included. We introduced our study and its aims to the upazila Family Planning Officer in Sakhipur, who introduced us to the local female Family Welfare Assistant. To select the participants, a list of women who were pregnant or had a recent birth ($\leq$1 year) was collected from the Family Welfare Assistant who also helped the research team to visit the participants in their home. Community health workers (Family Welfare Assistant, Family Welfare Visitor, Community Health Care Provider and Traditional Birth Attendant) were also interviewed to explore their experiences and local knowledge.

Data collection

We developed interview guides and translated those into Bangla and then pilot tested and adjusted where necessary. The major topics covered in the interview guides for the participants

were the perceptions on birthweight, identification and classification of newborns based on birthweight, factors perceived to affect newborn's birthweight, preventive practices and the care they perceived for LBW newborns and barriers to care for LBW newborns. We conducted 32 IDIs; with pregnant women (n = 11), recently delivered women (n = 12), mothers-in-law (n = 5) and husbands (n = 4). We conducted KIIs with the community health workers (n = 4), and two FGDs with the husbands with eight participants in each (Table 1). The IDIs and the FGDs were conducted by the lead author (MKN) and a research assistant with qualitative research expertise. All the KIIs were conducted by MKN. All the interviews and discussions were held in private locations preferred by participants. Each of the interviews and discussions lasted between 30-70 minutes and was digitally audio-recorded.

Data analysis

We used a thematic approach for analysing the data following several steps. All the interviews and FGDs were recorded and transcribed verbatim in Bangla. The transcripts and notes were then translated into English by the first author (MKN). Five transcripts were discussed with the other two investigators (AA and CRG) for quality check. Then MKN performed line by line manual coding and developed a list of codes based on the key topics included in the data collection guidelines. The codes were reviewed by AA to ensure inter-coder reliability. Then the text pertaining to each topic code was discussed among the researchers and summarised in a document that presented the findings for each theme using quotes and tables. Themes were adjusted and refined after several discussions among the members of the research team. Themes were triangulated using data from IDIs, FGDs and KIIs. For data presentation, we also selected quotations as examples of typical responses of the participants.

Ethics approval

Ethics approval was obtained from the institutional review board of the International Centre for Diarrhoeal Disease Research, Bangladesh (icddr,b) (PR -17055). We obtained informed written consent from all participants before the start of each interview and FGD.

Results

We identified six key themes to present the responses from all the participants - "recognition and importance of birthweight", "perceived role of birthweight on newborn's health status", "perceived causes of low and high birthweight", "attitudes towards prevention of LBW or

Table 1. Methods and sampling.

Methods of data collection	Type of respondents	Number
In-depth interviews		
	Pregnant women	11
	Recently delivered women (≤1 year)	12
	Mothers-in-law	5
	Husbands	4
Focus group discussions	Husbands	2<br>(n = 16)
Key informant interviews	Community health workers<br>(Family Welfare Assistant, Family Welfare Visitor, Community Health Care Provider and Traditional Birth Attendant)	4

https://doi.org/10.1371/journal.pone.0221691.t001

small birth size", "perceptions of care practices for LBW or small newborns" and "barriers to care-seeking for LBW or small newborns".

Demographic characteristics of the participants

There were four groups of participants in this study - women (pregnant and recently delivered women), mothers-in-law, husbands and community health workers. The age of the women ranged from 16 to 35 years, with the mean age of 23 years. Of the 23 women, most had attained primary education, however, about a fifth of the women did not have any formal education. No women were employed; excluding one who was a primary school teacher. The mean age of the mothers-in-law was 50 years (range 45 to 60 years). Of the five mothers-in-law, three did not have any formal education. The mean age of husbands was 28 years (range 22 to 39 years). The majority of the husbands had primary education or higher. The age of the interviewed community health workers ranged from 28 to 55 years (mean 40 years). Almost all of the women and their families lived with their extended family.

Recognition and importance of birthweight

A majority of the participants of all groups were unaware of their own newborns' birthweight. However, a few women who had a birth in a health facility knew their newborns' birthweight. In general, participants were not familiar with the technical terms of birthweight categories (e.g. LBW, high birthweight). Birth size was more recognised than birthweight and frequently used by most of the participants to describe different categories of birthweight. In addition to birth size, several participants used some other external physical descriptors (Table 2) to refer to different categories of birthweight, although substantial overlap existed across the categories. When we prompted the participants to classify newborn's birthweight based on those physical descriptors including birth size, most of them classified birthweight into four categories - very LBW, LBW, normal birthweight and high birthweight, only a few participants mentioned very high birthweight (Table 2), however, a few women and mothers-in-law were using same physical descriptors to refer to the newborns categorised as LBW and very LBW (e.g. "skinny", "wrinkled-skinned"); or normal and high birthweight (e.g. "healthy", "well-nourished"). These classifications were mostly based on previous observations of newborns at birth, holding them and comparing the newborn's birthweight or birth size with the birthweight or birth size of a newborn who was being weighed in hospital. *"We can easily understand the baby's birth size by seeing or holding."* (Recently delivered woman, R023).

Most participants referred to the LBW newborns as being small in size, thin, weak, skinny, malnourished and wrinkled-skinned in appearance. Further, some participants considered very LBW newborns as more fragile than the LBW newborns and some compared these very

Table 2. Recognition of different categories of birthweight and physical descriptors of these categories.

Birthweight categories	Birth size categories	Other physical descriptors
Very LBW	"Very small in size"	"Weighs very low", "weighs like a bird or doll", "very weak", "skinny", "looks ill", "extremely malnourished", "wrinkled-skinned", "unable to open eyes"
LBW	"Small in size"	"Weighs low", "thin", "weak", "skinny", "malnourished", "wrinkled-skinned"
Normal birthweight	"Medium in size"	"Looks natural", "healthy", "well-nourished", "ideal birthweight", "average birthweight"
High birthweight	Large in size	"Fat", "big", "weighs heavy", "weighs like a pumpkin", "healthy", "well-nourished", "over-nourished"

https://doi.org/10.1371/journal.pone.0221691.t002

LBW newborns to a "bird" or "doll". A normal birthweight newborn was described as medium or average in size, natural looking, well-nourished and an ideal birthweight by most participants. Further, high birthweight newborns were often referred as being large in size, fat, big, heavy, like a pumpkin, and both healthy and over-nourished (Table 2). These interpretations of birthweight categories have been used throughout our paper to document families' perceptions and practices related to birthweight.

Most participants acknowledged that they did not discuss the birthweight of their newborns before or after birth. With regard to the reason for this, several women and mothers-in-law expressed their lack of interest in measuring birthweight. *"No, I did not discuss my baby's birthweight. I don't think it is needed to be measured."* (Recently delivered woman, R018).

In general, after a birth, most of the participants were more concerned with the skin colour, appearance and sex of the newborn, rather than the birthweight. In some cases, extra emphasis was placed by the participants for giving birth to a male infant over an infant with normal birthweight as reflected in the quote of a recently delivered woman: "*We were happy that the baby was a boy. Birthweight was not important to us.*" (Recently delivered woman, R012).

Community health workers who were the main sources of health-related information for community members, also confirmed that there was no discussion of birthweight in the rural community. "*No, no one talked to us on newborn's birthweight. No one asked any question on birthweight. I never faced any question like that - how much my baby will weigh.*" (Community health worker, C052).

Perceived role of birthweight on newborn's health status

Most participants did not consider birthweight an indicator of the health status of a newborn. Although there was a desire for a healthy newborn among the participants, the majority of the participants felt no necessity of measuring birthweight to assess their newborn's health status.

Instead, the perceived criteria of a healthy newborn included crying right after birth, having a "normal shape" and movement of the limbs, free from illnesses, able to suck and able to defecate; while the perceived criteria of an ill newborn included not breathing properly, not crying, poor limb movement, unable to suck, unable to defecate, scalp swelling, suffering from illnesses such as cold, fever and diarrhoea. For instance, a recently delivered woman noted, "*When a baby is born, if it cries immediately after birth, it is a healthy baby. When we see a baby does not cry at birth, it means that the baby is at pain, it is not healthy, it is sick.*" (Recently delivered woman, R021).

Another issue that we explored under this theme was whether the participants could associate any risk with a LBW or small birth size newborn in relation to newborn's health status. While a few participants expressed their unawareness of the risk associated with LBW, several participants indicated no adverse consequences associated with LBW, if the newborn was healthy by appearance. "*It is not always true that a LBW baby will have a problem. If the baby looks healthy but weighs less, it will be alright. All babies will grow up eventually irrespective of weighing less or more at birth. Not everyone is born with the same weight, some are born with less weight, and some are born with more weight. They will grow up in different stages of their lives.*" (Pregnant woman, P005). However, with regard to a very LBW newborn, a few participants expressed a sense of relatedness to some illnesses such as - fever, cold, pneumonia, jaundice, respiratory distress, limb paralysis, brain diseases and rashes.

Perceived causes of low and high birthweight

Participants reported a number of causes for both LBW and high birthweight (Table 3). Most of the causes were related to maternal health and maternal behaviour. Poor maternal nutrition

and inadequate diet in pregnancy were mostly considered causes of LBW by most women and their families. Further, maternal excessive food and water consumption during pregnancy were also perceived by some mothers-in-law as causes of LBW. According to these mothers-in-law, excessive food or water consumption by a pregnant woman would allow less space in the womb which would eventually compress the fetus in the womb and lead to the birth of a small newborn. This perception was also reflected among a few women as quoted below, "*I heard that if a mother eats a lot during pregnancy, though she gets adequate nutrition, there is a lack of space in the mother's womb. Because of that, I heard that the baby does not get enough space to grow in the womb which keeps baby's size small.*" (Recently delivered woman, R024).

Another common cause of LBW included maternal short stature. Several mothers-in-law and a few of women expressed that maternal small stature was a reason for giving birth to a LBW newborn. This belief was supported by one community health worker who reported that the families' belief that parental stature was the main determinant of the birth size of the newborn.

Further, a few participants referred to the influence of supernatural spirit and sins in the previous life as reasons for giving birth to a LBW newborn. For instance, one husband of a recently delivered woman remarked, "*God gives us baby. Sometimes a weak baby is born with a lot of birth defects. Not every weak baby is born because of maternal food habit, some of them are born weak and sick because of their sins in the previous life.*" (Husband, H040).

A small number of women associated the female sex of the newborn with LBW. For instance, one recently delivered woman explained as follows, *"I was able to eat a lot during giving birth to my girl baby but the baby was small in size. But this time when I gave birth to a boy baby, I could not eat at all but my son's birth size was large. My husband told me that the male baby is usually born with large size."* (Recently delivered woman, R014).

The other common causes of LBW included maternal illnesses during pregnancy, anaemia, giving birth to twins, excessive household chores in pregnancy and preterm delivery. In contrast, several participants associated maternal over-nutrition, overweight, excessive food consumption during pregnancy, and reduced movement during pregnancy, with a high birthweight newborn. It was perceived that pregnant women who reduced their household chores and who took too much rest had high birthweight newborns (Table 3).

Table 3. Summary of perceived causes of low and high birthweight.

Low birthweight	High birthweight
-Poor maternal nutrition<br>-Inadequate diet in pregnancy<br>-Mothers having less space in the womb because of excessive food and water consumption in pregnancy<br>-Maternal anaemia<br>-Maternal short stature<br>-Maternal small size uterus<br>-Mothers being too thin<br>-Maternal young age<br>-Maternal illnesses during pregnancy<br>-Mother having an infection in the blood<br>-Maternal weakness<br>-Lack of care in pregnancy<br>-Excessive household chores in pregnancy<br>-Mother not taking iron tablets during pregnancy<br>-Prolonged labour pain<br>-Preterm delivery<br>-Giving birth to twins<br>-Giving birth to a female infant<br>-Sins in the previous life<br>-Supernatural power<br>-God's will	-Maternal over-nutrition<br>-Maternal overweight<br>-Maternal excessive food consumption during pregnancy<br>-Caesarean section<br>-Maternal reduced movement during pregnancy<br>-Mother having a large space in the womb because of very less food consumption in pregnancy<br>-Giving birth to a male infant<br>-Mother wearing loose clothes during pregnancy<br>-Reduced household chores in pregnancy

https://doi.org/10.1371/journal.pone.0221691.t003

Attitudes towards prevention of LBW or small birth size

Women's practices to prevent a LBW infant were influenced by their perceptions of causes and risk of LBW, most of which often discouraged them to perform preventive practices in pregnancy. For instance, preventive practices to LBW such as maternal diet were restricted by a common belief of most participants that giving birth to a small size or LBW newborn would avoid pregnancy complications, caesarean section and facility delivery. A recently delivered woman noted, *"I think it is good to give birth to a LBW baby as it can help to have a normal delivery. Once the baby is born it can get nutrition from the food. The baby can put on weight as it grows."* (Recently delivered woman, R015).

Mothers-in-law were the main sources of such belief who felt giving birth to a normal or high birthweight newborn could lead a mother to have delivery complications, including caesarean section or death. "*My grandson was small in size at birth. I was happy with my grandson's birth size. By the grace of God, we did not have to go to the hospital, the delivery occurred at home. If my grandson was large in birth size, we would have to go to the hospital, the mother would need a caesarean section.*" (Mother-in-law, M028).

Many women whilst pregnant decreased food intake and avoided eating nutritious food to give birth to a small newborn, in order to have an easier delivery and to avoid a caesarean section. *"I want to have a normal delivery. If I eat a lot in pregnancy that would increase the baby's size in my womb, which would lead to a caesarean section. It might put my life at risk."* (Pregnant woman, P007).

Another belief that maternal short stature was the main determinant of small birth size newborn discouraged women to perform good practices in pregnancy such as attending antenatal care. One recently delivered woman with short stature in this regard noted, "*This is very normal that our baby is small in size. All of my sisters had a small size baby. Because we all are short and thin. Our babies will be small no matter what we do in pregnancy.*" (Recently delivered woman, R023).

Further, several participants expressed their unawareness on the preventive practices to LBW. A few husbands in FGDs expressed their lack of awareness of an ideal portion of food for pregnant women. "*One of my concerns is - we eat without knowing the ideal amount of food. We do not know the amount of food needed for a pregnant mother. We live in a village. We know she needs nutritious food these days. . . But we do not know what is the appropriate amount of those food needed for her and baby's well-being. We think that if she eats well, she and the baby will be fine. But we do not know the exact dose.*" (Husband, FGD-2).

As a reason of the unawareness, a few participants indicated that there was a lack of information from community health workers on birthweight. Community health workers interviewed in this study also agreed, acknowledging that birthweight was not usually discussed while they counselled the families about good practices to ensure a healthy newborn. "*Truly speaking we do not talk about birthweight directly. We tell the mothers that if you eat nutritious food, your baby will be healthy. Actually, birthweight was not specifically discussed. I won't lie. I never talked about the measurement of ideal birthweight with the mothers.*" (Community health worker, C051).

Perceptions of care practices for LBW or small newborns

In general, the majority of participants felt that no additional treatment was needed for a LBW newborn who appeared healthy by physical appearance as reflected in a quote of a mother-in-law, "*There is no problem if a baby is born with LBW. This can be treated with a doctor's suggestions and giving enough food.*" (Mother-in-law, M025).

However, a few participants reported extra care practices for the very small or LBW newborn who appeared to be unwell including breastfeeding, providing supplemental formula,

seeking health care provider's advice, seeking spiritual treatment, oil massage and keeping the infant warm.

The majority of the participants reported breastfeeding as the main treatment for a LBW or small size newborn and to feed the mother well to ensure nutritious breastmilk. Although several women emphasised early initiation of breastfeeding for a LBW newborn; some recently delivered women shared their experience of delaying breastfeeding for their small newborns for 2 to 3 days after birth. The practice of delayed initiation of breastfeeding was attributable to a number of reasons, including a perceived lack of breastmilk and families' perceived need for additional food for the unwell newborn. In the interim, small newborns were reported to be fed animal (cow and goat) milk, sugary water or honey using a cotton ball at short intervals as a treatment to improve the newborn's health. "*The sugar was mixed with water and was fed to the newborn using a cotton ball or a small stick. The baby then sucked it. It kept my baby's throat moist. If the throat is moist, the baby remains healthy.*" (Recently delivered woman, R016). Further, providing supplemental formula was a common way to treat a small size newborn. In this context, a father of twin infants remarked, "*The demand of breastmilk for both of my twin babies cannot be fulfilled at the same time. So, I bought formula milk from outside for my babies.*" (Husband, H038).

Several participants felt the need for seeking health care provider's advice for the newborns who were extremely small and unwell. However, spiritual treatment by a religious figure was also regarded as a treatment of a very LBW or very small newborn who appeared ill. Other care practices for a LBW or small newborn included oil (mustard oil) massage, keeping the small newborn warm using wraps (cotton cloths), feeding with saline, vitamins and traditional medicine including herbs, keeping the house clean and bathing the newborn with warm water.

Barriers to care-seeking practices for LBW or small newborns

Common factors which limited the families' care-seeking practices for a LBW newborn included lack of decision-making power by women, superstition, financial constraints, home birth, and lack of resources in the facility.

We found that maternal lack of a decision-making power was an important barrier which impacted the care of their small and unwell newborn received. The decision to seek care for a newborn was usually either by the husband or sometimes by the mother-in-law. According to several women, their husbands were not involved in any care practice of the newborn at home and the mothers-in-law, because of their traditional beliefs were more likely to discourage the women for care-seeking from the health facility unless the newborn was severely ill, which often constrained the care-seeking for a LBW newborn. As quoted below, because of lack of a decision-making power, a recently delivered woman did not get an opportunity to go to the health facility with her small and unwell newborn and consequentially some suggestions from the doctors for her newborn remained unknown to the mother which impacted the care practices of the unwell newborn, "*I usually do not go outside or to the doctors. For the treatment of my weak newborn, sometimes my husband and sometimes my mother-in-law went to the doctors. They decided when to go to the doctor for my baby. But this does not help always because I am the only person at home who takes care of my baby. When my husband came back home from the hospital, he did not say the things in detail which needed to be done for my weak baby.. . . I had to ask him several times about the doctor's advice.*" (Recently delivered woman, R017).

Superstition also posed a great challenge for care-seeking behaviour of the rural families for a LBW newborn. One mother-in-law and one husband (of Hindu religion) reported protecting their small newborns from "evil's eyes" or "bad spirit" for approximately seven to 45 days by

isolating the newborn with the mother inside a separate room, resulting in delayed care-seeking. "*In our religion baby needs to be kept in a separate room with its mother for at least seven to nine days. My granddaughter was in a separate room for seven days. During that time only a few people were allowed to enter the room. This is important for us to keep the baby safe from bad spirit.*" (Mother-in-law, M027).

Furthermore, a few recently delivered women reported an inability to buy nutritious food while breastfeeding their small newborn. These poorer women and their families were also unable to access a health facility for their small and unwell newborn, whereas families who were relatively wealthier were more likely to seek care from a health facility if the newborn was unwell. Community health workers reported a lack of resources; such as lack of staff and lack of infrastructure to treat a LBW newborn at the community level health facility.

Discussion

Our study found that birthweight was not well-recognised among women and their families in this rural community, and was not considered a priority for assessing a newborn's health status. Perceptions on causes of low and high birthweight were different although tangled together. LBW was not considered as an adverse health outcome of a newborn unless the newborn appeared unwell. A lack of awareness existed for preventive practices to LBW which were predominantly restricted by a common belief that giving birth to a small newborn could avoid pregnancy complications and caesarean section. As an effort to avoid caesarean section during birth, women tended to perform potentially harmful practices in order to give birth to a small size newborn; such as avoiding nutritious food and decreasing food intake in pregnancy. Most of the participants regarded breastfeeding as a major treatment for a LBW newborn however, for several LBW newborns, their breastfeeding commenced up to 2 to 3 days after birth. Maternal lack of decision-making power and superstition were major challenges for the care of a LBW newborn in the rural community.

There was limited knowledge of birthweight and the risk associated with LBW in the rural community. Although women and their families seemed concerned about their newborns' health status, most did not consider birthweight as an indicator of the newborn's health status; which is in accord with the findings of a study conducted in rural India [29]. We also found that instead of birthweight, assessment of the health status of a newborn frequently included observable criteria such as crying, movement of the limbs at birth and being able to suck, which are in line with prior research [29, 34]. Further, classifying newborn's birthweight into different categories was subjective and often included different physical descriptors of the newborns with considerable overlap across the categories. Several participants did not consider LBW or very LBW as a criterion of a newborn's illness unless the newborn appeared unwell which is similar to a previous study conducted in Uganda [35]. As a result, LBW newborns were not likely to receive timely and proper care and hence were vulnerable to adverse health outcomes. According to Marsh et al.'s framework, the care for a LBW newborn commences with recognition [36]. Therefore, the delay in LBW recognition needs to be addressed in the rural community through formulating socio-culturally appropriate interventions which focus on the easy identification of LBW newborns. Involving community health workers for counselling the families about recognition of ideal birthweight and the importance of recognising LBW could be a useful approach.

The commonly perceived causes of LBW included maternal inadequate diet, poor nutrition, and illnesses in pregnancy, preterm delivery, and twin births, which are in line with the findings of previous research in similar settings [30, 37]. This suggests that most women and their families were aware of several important risk factors of the newborn's poor birthweight,

however, there were several other causes reported which may put their newborn's health at risk by limiting the preventive practices of LBW. For instance, the influence of supernatural spirit was sometimes cited as a cause of giving birth to a LBW newborn which is consistent with a few prior studies [30, 35]. A previous study suggests that this belief sometimes prevents pregnant women from seeking antenatal care as they remain at home and avoid travelling to the hospital to keep themselves safe from the bad spirit [38]. The important role of such belief has also been reflected in our findings in terms of care practices of a LBW newborn in the rural community, where we found that spiritual treatment by the religious or spiritual leader was a common approach to improve small newborn's health. This belief of supernatural spirit needs to be addressed by implementing interventions that improve awareness in appropriate care-seeking practices in the rural communities, as our study also identified such belief as an important barrier to care-seeking for a LBW newborn. Another approach could be educating and training spiritual healers to encourage the families to seek care for their small newborns from the formal care providers. One further perceived cause of giving birth to a LBW newborn was maternal short stature which was considered a determinant of poor fetal growth. Although maternal stature has an impact on fetal growth [39], we found this deterministic attitude as a barrier to performing preventive practices of LBW such as - attending antenatal care. There is a need to sensitise families about the risk factors of LBW through community mobilisation and group counselling to change this deterministic attitude.

Despite considering maternal inadequate diet in pregnancy as a reason of LBW, a fear of caesarean section often led the women to eat less and avoid nutritious food in pregnancy. The practice is consistent with prior studies conducted in South Asian countries including Bangladesh [40, 41]. We found that due to this belief many women and mothers-in-law expressed their preference for a LBW newborn to avoid delivery complications and caesarean section, although most of them concurred with the fact that "normal birthweight" is an ideal birthweight. In addition, we found that the preventive practices to LBW related to maternal diet were limited by the belief that women's increased food consumption was a cause for giving birth to a LBW newborn. In rural Bangladesh, where nearly a quarter of women of reproductive age are underweight [31], such harmful beliefs regarding maternal diet in pregnancy should not be overlooked. Health care programs should include information on the importance of a nutritious and adequate diet in pregnancy through community-level education.

Similar to a prior cross-sectional study [42], there was a lack of awareness among the participants regarding the preventive practices to LBW. However, in our study, several participants attributed this to a lack of counselling by the community health workers; which was later acknowledged by the community health workers. Importantly, a few husbands raised an issue of their lack of awareness with the ideal food portions required in pregnancy for the well-being of the developing infant. This finding highlights an important consideration for program managers of nutritional education, who need to include education of food portions required for an adequate maternal diet. This was a key part of an intervention recently trialled in Bangladesh [43].

The World Health Organization recommends early and exclusive breastfeeding to improve the care and survival of LBW newborns [44]. Our findings partly conform to this recommendation as breastfeeding was frequently used by the participants as a treatment of LBW newborns. However, in terms of initiation of breastfeeding, there were contrasting findings; for several LBW newborns, their breastfeeding commenced up to 2 to 3 days after birth. Animal (cow and goat) milk, sugary water, honey and formula were the reported substitutes during this period. Although our study did not specifically examine the reasons for this practice, we found that a lack of adequate breastmilk and families' perceived need for additional food for the unwell newborn mainly contributed to this practice. A similar practice has been echoed in

a study conducted in Bangladesh on overall newborn care practices, where newborns were breastfed only after 3 to 5 days [45]. Delay in the initiation of breastfeeding is associated with a higher risk of newborn mortality [46], it is, therefore, essential to counsel women and their families in rural communities of the advantages of early initiation of breastfeeding for their newborn's health. As in a prior study in rural Bangladesh [45], we found that massage with mustard oil was viewed as a care practice of an unwell small newborn. Also, in our study, the concept of keeping the small newborn warm was also reported by a few participants as a care practice of small newborns which is in line with a prior study conducted in Zambia [47]. Our study revealed that the care-seeking behaviour for the LBW newborn's well-being was often limited by a maternal lack of decision-making power. This could be illustrated by the fact that in our study, most of the interviewed women were from an extended family where their husbands and mothers-in-law were the main decision makers [48, 49]. In rural Bangladesh, women play the main role of carer for their children, and most of the husbands usually do not participate in any care practices for their children, thus most of them are unacquainted with their newborn's health status. Further, in our study, mothers-in-law held traditional beliefs on the care practices of a newborn which potentially were likely to limit the proper care-seeking practices for a LBW newborn. In rural Bangladesh where women's poor decision-making power for health care-seeking is an existing problem [49], health education emphasising the care practices for a LBW newborn could be provided at the family level involving women, their husbands and the mothers-in-law in order to improve the survival of LBW newborns.

Strengths and limitations

A strength of this study is that we triangulated our findings using perspectives from different community members. Also, we included participants from different ages, genders and relationship with the infant which allowed us to identify similarities and differences between shared beliefs and values across community members.

We acknowledge several limitations of our study that should be considered while interpreting our findings. First, the selection process whereby women and the families were identified by the Family Welfare Assistant who was known to her and might lead to selection bias. Second, we did not purposively interview families who had a low birthweight infant as a majority of the families were unaware of their own infants' birthweight due to home birth. Third, some part of our findings were based on retrospective reports of practices of the women and their families around childbirth and may be subject to recall bias. However, our triangulation approach has minimised these potential weaknesses.

Conclusions

Our study found that birthweight was not well-recognised and often excluded from the assessment criteria of a newborn's health status in rural Bangladesh. The perceived causes and risks of LBW were associated with several beliefs in the rural community, which highlighted substantial challenges to LBW prevention and care practices. Preventive practices concerning maternal diet in pregnancy were often compromised by several misconceptions which often led the pregnant women to perform some harmful practices. Our findings have important implications for program planners, who could incorporate a focus on recognition of birthweight and associated perceptions and practices into all health policies, strategies and programs developed for improving newborn's birthweight in rural settings of Bangladesh and other similar settings by addressing local socio-cultural beliefs and practices.

Supporting information

S1 File. Interview guide for Birthweight study IDI_English.
(PDF)

S2 File. Interview guide for Birthweight study IDI_Bangla.
(PDF)

S3 File. Interview guide for Birthweight study FGD_English.
(PDF)

S4 File. Interview guide for Birthweight study FGD_Bangla.
(PDF)

S5 File. Interview guide for Birthweight study KII_English.
(PDF)

S6 File. Interview guide for Birthweight study KII_Bangla.
(PDF)

Acknowledgments

We are grateful to all the participants of this study for their valuable time, generosity and cooperation. We would like to thank Basanti Sannyasi who assisted in the data collection of this study.

Author Contributions

Conceptualization: Monjura Khatun Nisha, Camille Raynes-Greenow, Ashraful Alam.

Data curation: Monjura Khatun Nisha, Aminur Rahman.

Formal analysis: Monjura Khatun Nisha, Camille Raynes-Greenow, Ashraful Alam.

Investigation: Monjura Khatun Nisha, Camille Raynes-Greenow, Aminur Rahman, Ashraful Alam.

Methodology: Monjura Khatun Nisha, Camille Raynes-Greenow, Ashraful Alam.

Project administration: Monjura Khatun Nisha.

Resources: Monjura Khatun Nisha, Camille Raynes-Greenow, Aminur Rahman, Ashraful Alam.

Software: Monjura Khatun Nisha.

Supervision: Monjura Khatun Nisha, Camille Raynes-Greenow, Aminur Rahman, Ashraful Alam.

Validation: Monjura Khatun Nisha, Ashraful Alam.

Visualization: Ashraful Alam.

Writing – original draft: Monjura Khatun Nisha.

Writing – review & editing: Monjura Khatun Nisha, Camille Raynes-Greenow, Aminur Rahman, Ashraful Alam.

References

1. World Health Organization. International Statistical Classification of Diseases and Related Health Problems - 10th revision. Instruction manual. Geneva: World Health Organization, 2011.

2. Wilcox AJ. On the importance–and the unimportance–of birthweight. International journal of epidemiology. 2001; 30(6):1233–41. Epub 2002/02/01. https://doi.org/10.1093/ije/30.6.1233 PMID: 11821313.
3. Kramer MS. The Epidemiology of Adverse Pregnancy Outcomes: An Overview. The Journal of Nutrition. 2003; 133(5):1592S–6S. https://doi.org/10.1093/jn/133.5.1592S PMID: 12730473
4. Wardlaw TM. Low birthweight: country, regional and global estimates: UNICEF; 2004.
5. UNICEF (2009) The state of the world's children 2009: maternal and newborn health: United Nations Children's Fund.
6. Black RE, Allen LH, Bhutta ZA, Caulfield LE, de Onis M, Ezzati M, et al. Maternal and child undernutrition: global and regional exposures and health consequences. The Lancet. 2008; 371(9608):243–60. https://doi.org/10.1016/S0140-6736(07)61690-0.
7. Wilcox AJ. On the importance—and the unimportance— of birthweight. International Journal of Epidemiology. 2001; 30(6):1233–41. https://doi.org/10.1093/ije/30.6.1233 PMID: 11821313
8. Christian P, Lee SE, Donahue Angel M, Adair LS, Arifeen SE, Ashorn P, et al. Risk of childhood undernutrition related to small-for-gestational age and preterm birth in low- and middle-income countries. International journal of epidemiology. 2013; 42(5):1340–55. Epub 2013/08/08. https://doi.org/10.1093/ije/dyt109 PMID: 23920141; PubMed Central PMCID: PMC3816349.
9. Huang C, Martorell R, Ren A, Li Z. Cognition and behavioural development in early childhood: the role of birth weight and postnatal growth. International Journal of Epidemiology. 2013; 42(1):160–71. https://doi.org/10.1093/ije/dys207 PMC3600622. PMID: 23243117
10. Dalziel SR, Parag V, Rodgers A, Harding JE. Cardiovascular risk factors at age 30 following pre-term birth. International Journal of Epidemiology. 2007; 36(4):907–15. https://doi.org/10.1093/ije/dym067 PMID: 17468503
11. Huxley R, Owen CG, Whincup PH, Cook DG, Colman S, Collins R. Birth weight and subsequent cholesterol levels: exploration of the "fetal origins" hypothesis. Jama. 2004; 292(22):2755–64. Epub 2004/12/09. https://doi.org/10.1001/jama.292.22.2755 PMID: 15585736.
12. Huxley R, Neil A, Collins R. Unravelling the fetal origins hypothesis: is there really an inverse association between birthweight and subsequent blood pressure? Lancet (London, England). 2002; 360 (9334):659–65. Epub 2002/09/21. https://doi.org/10.1016/s0140-6736(02)09834-3 PMID: 12241871.
13. Phillips DI, Walker BR, Reynolds RM, Flanagan DE, Wood PJ, Osmond C, et al. Low birth weight predicts elevated plasma cortisol concentrations in adults from 3 populations. Hypertension (Dallas, Tex: 1979). 2000; 35(6):1301–6. Epub 2000/06/17. https://doi.org/10.1161/01.hyp.35.6.1301 PMID: 10856281.
14. World Health Organization. Comprehensive implementation plan on maternal, infant and young child nutrition. 2014.
15. WHO U. Low birthweight: country, regional and global estimates. Geneva, UNICEF and WHO. 2004.
16. World Health Organisation. World Health Assembly WHA Global Nutrition Targets 2025; Low birth weight policy brief. Geneva: World Health Organisation; 2014.
17. Lee AC, Kozuki N, Cousens S, Stevens GA, Blencowe H, Silveira MF, et al. Estimates of burden and consequences of infants born small for gestational age in low and middle income countries with INTERGROWTH-21st standard: analysis of CHERG datasets. BMJ. 2017; 358. https://doi.org/10.1136/bmj.j3677 PMID: 28819030
18. Walton KA, Murray LJ, Gallagher AM, Cran GW, Savage MJ, Boreham C. Parental recall of birthweight: A good proxy for recorded birthweight? European Journal of Epidemiology. 2000; 16(9):793–6. https://doi.org/10.1023/a:1007625030509 PMID: 11297220
19. Catov JM, Newman AB, Kelsey SF, Roberts JM, Sutton-Tyrrell KC, Garcia M, et al. Accuracy and Reliability of Maternal Recall of Infant Birth Weight Among Older Women. Annals of Epidemiology. 2006; 16 (6):429–31. https://doi.org/10.1016/j.annepidem.2005.09.004 PMID: 16280248
20. Shakya KL SN, Bhatt MR, Hepworth S, Onta SR. Accuracy of low birth weight as perceived by mothers and factors influencing it: a facility based study in Nepal. Int J Med Res Health Sci. 2015; 4(2):274–80. https://doi.org/10.5958/2319-5886.2015.00051.X
21. Lule SA, Webb EL, Ndibazza J, Nampijja M, Muhangi L, Akello F, et al. Maternal recall of birthweight and birth size in Entebbe, Uganda. Tropical medicine & international health: TM & IH. 2012; 17 (12):1465–9. Epub 2012/09/22. https://doi.org/10.1111/j.1365-3156.2012.03091.x PMID: 22994260; PubMed Central PMCID: PMC3627817.
22. Araujo CL, Dutra CL, Hallal PC. Validity of maternal report on birth weight 11 years after delivery: the 1993 Pelotas Birth Cohort Study, Rio Grande do Sul State, Brazil. Cadernos de saude publica. 2007; 23 (10):2421–7. Epub 2007/09/25. https://doi.org/10.1590/s0102-311x2007001000017 PMID: 17891302.

23. Li CY, Wei JN, Lu TH, Chuang LM, Sung FC. Mothers tended to overreport categorical infant birth weight of their children. Journal of clinical epidemiology. 2006; 59(12):1319–25. Epub 2006/11/14. https://doi.org/10.1016/j.jclinepi.2006.02.018 PMID: 17098575.

24. Mbuagbaw L, Gofin R. Can recall of birth size be used as a measure of birthweight in Cameroon? Paediatric and perinatal epidemiology. 2010; 24(4):383–9. Epub 2010/07/14. https://doi.org/10.1111/j.1365-3016.2010.01128.x PMID: 20618728.

25. Blanc AKaW T Monitoring low birth weight: An evaluation of international estimates and an updated estimation procedure. Bulletin of the World Health Organization,. 2005; 83:178–85. PMID: 15798841

26. Channon AA. Can mothers judge the size of their newborn? Assessing the determinants of a mother's perception of a baby's size at birth. Journal of biosocial science. 2011; 43(5):555–73. Epub 2011/06/17. https://doi.org/10.1017/S0021932011000198 PMID: 21676278.

27. Aruldas K, Kant A, Mohanan PS. Care-seeking behaviors for maternal and newborn illnesses among self-help group households in Uttar Pradesh, India. Journal of health, population, and nutrition. 2017; 36 (Suppl 1):49–. https://doi.org/10.1186/s41043-017-0121-1 PMID: 29297413.

28. Hill Z, Kendall C, Arthur P, Kirkwood B, Adjei E. Recognizing childhood illnesses and their traditional explanations: exploring options for care-seeking interventions in the context of the IMCI strategy in rural Ghana. Tropical Medicine & International Health. 2003; 8(7):668–76. https://doi.org/10.1046/j.1365-3156.2003.01058.x PMID: 12828551

29. Darmstadt GL, Kumar V, Yadav R, Shearer JC, Baqui AH, Awasthi S, et al. Community perceptions of birth weight in rural Uttar Pradesh, India: implications for care of low-birth-weight infants. Journal of perinatology: official journal of the California Perinatal Association. 2008; 28 Suppl 2:S53–60. Epub 2008/12/17. https://doi.org/10.1038/jp.2008.168 PMID: 19057569.

30. Koenraads M, Phuka J, Maleta K, Theobald S, Gladstone M. Understanding the challenges to caring for low birthweight babies in rural southern Malawi: a qualitative study exploring caregiver and health worker perceptions and experiences. BMJ Global Health. 2017; 2(3):e000301. https://doi.org/10.1136/bmjgh-2017-000301 PMC5656136. PMID: 29082008

31. National Institute of Population Research and Training (NIPORT) MaA, and ICF International. Bangladesh Demographic and Health Survey 2014. Dhaka, Bangladesh, and Rockville, Maryland, USA: NIPORT, Mitra and Associates, and ICF International, 2016.

32. Arifeen SE, Mullany LC, Shah R, Mannan I, Rahman SM, Talukder MRR, et al. The effect of cord cleansing with chlorhexidine on neonatal mortality in rural Bangladesh: a community-based, cluster-randomised trial. The Lancet. 2012; 379(9820):1022–8. https://doi.org/10.1016/S0140-6736(11)61848-5.

33. Yaya S, Bishwajit G, Ekholuenetale M, Shah V. Awareness and utilization of community clinic services among women in rural areas in Bangladesh: A cross-sectional study. PLoS ONE. 2017; 12(10): e0187303. https://doi.org/10.1371/journal.pone.0187303 PMC5659786. PMID: 29077754

34. Alam A, Rasheed S, Khan NU, Sharmin T, Huda TM, Arifeen SE, et al. How can formative research inform the design of an iron-folic acid supplementation intervention starting in first trimester of pregnancy in Bangladesh? BMC Public Health. 2015; 15(1):374. https://doi.org/10.1186/s12889-015-1697-2 PMID: 25887449

35. Nabiwemba EL, Atuyambe L, Criel B, Kolsteren P, Orach CG. Recognition and home care of low birth weight neonates: a qualitative study of knowledge, beliefs and practices of mothers in Iganga-Mayuge Health and Demographic Surveillance Site, Uganda. BMC Public Health. 2014; 14(1):546. https://doi.org/10.1186/1471-2458-14-546 PMID: 24888464

36. Marsh DR, Darmstadt GL, Moore J, Daly P, Oot D, Tinker A. Advancing Newborn Health and Survival in Developing Countries: A Conceptual Framework. Journal Of Perinatology. 2002; 22:572. https://doi.org/10.1038/sj.jp.7210793 PMID: 12368975

37. Imdad A, Bhutta ZA. Nutritional Management of the Low Birth Weight/Preterm Infant in Community Settings: A Perspective from the Developing World. The Journal of Pediatrics. 2013; 162(3, Supplement): S107–S14. https://doi.org/10.1016/j.jpeds.2012.11.060.

38. Finlayson K, Downe S. Why Do Women Not Use Antenatal Services in Low- and Middle-Income Countries? A Meta-Synthesis of Qualitative Studies. PLoS Medicine. 2013; 10(1):e1001373. https://doi.org/10.1371/journal.pmed.1001373 PMC3551970. PMID: 23349622

39. Sinha B, Taneja S, Chowdhury R, Mazumder S, Rongsen-Chandola T, Upadhyay RP, et al. Low-birth-weight infants born to short-stature mothers are at additional risk of stunting and poor growth velocity: Evidence from secondary data analyses. Maternal & Child Nutrition. 2018; 14(1):e12504. https://doi.org/10.1111/mcn.12504 PMID: 28840655

40. Ara S, Mominul IM, Kamruzzaman M, Toufiq EM, Sk S, Sabir HM. Assessment of social, economic and medical determinant of safe motherhood in Dhaka City: a cross-sectional study. American Journal of Life Science. 2013; 1(3):93–7.

41. Hutter I. Reduction of food intake during pregnancy in rural south India. Tropical medicine & international health: TM & IH. 1996; 1(3):399–405. Epub 1996/06/01. https://doi.org/10.1046/j.1365-3156.1996.d01-53.x PMID: 8673846.

42. Chowdhury A, Halder K, Haque I, Muhammad F, Hasan M. Status of Knowledge on the Risk Factors of Low Birth Weight among the Women of Reproductive Age in Rural Bangladesh. Epidemiology (Sunnyvale). 2017; 7(292):2161–1165.1000292.

43. Chowdhury M, Dibley MJ, Alam A, Kelly PJ, Afsana K, Raynes-Greenow C. The impact of an antenatal balanced plate nutrition education intervention on infant birthweight: a cluster randomised controlled trial in rural Bangladesh. Unpublished. 2018.

44. World Health Organization. Optimal feeding of low birthweight infants in low-and middle-income countries World Health Organization, 2011.

45. Winch PJ, Alam MA, Akther A, Afroz D, Ali NA, Ellis AA, et al. Local understandings of vulnerability and protection during the neonatal period in Sylhet district, Bangladesh: a qualitative study. The Lancet. 2005; 366(9484):478–85. https://doi.org/10.1016/S0140-6736(05)66836-5.

46. Smith ER, Hurt L, Chowdhury R, Sinha B, Fawzi W, Edmond KM, et al. Delayed breastfeeding initiation and infant survival: A systematic review and meta-analysis. PLOS ONE. 2017; 12(7):e0180722. https://doi.org/10.1371/journal.pone.0180722 PMID: 28746353

47. Sacks E, Moss WJ, Winch PJ, Thuma P, van Dijk JH, Mullany LC. Skin, thermal and umbilical cord care practices for neonates in southern, rural Zambia: a qualitative study. BMC pregnancy and childbirth. 2015; 15:149–. https://doi.org/10.1186/s12884-015-0584-2 PMID: 26177637.

48. Story WT, Burgard SA, Lori JR, Taleb F, Ali NA, Hoque DE. Husbands' involvement in delivery care utilization in rural Bangladesh: A qualitative study. BMC Pregnancy and Childbirth. 2012; 12(1):28. https://doi.org/10.1186/1471-2393-12-28 PMID: 22494576

49. Story WT, Burgard SA. Couples' reports of household decision-making and the utilization of maternal health services in Bangladesh. Social Science & Medicine. 2012; 75(12):2403–11. https://doi.org/10.1016/j.socscimed.2012.09.017.
